# Combinatorial Fusion Rules to Describe Codon Assignment in the Standard Genetic Code

**DOI:** 10.3390/life11010004

**Published:** 2020-12-23

**Authors:** Alexander Nesterov-Mueller, Roman Popov, Hervé Seligmann

**Affiliations:** 1 Institute of Microstructure Technology, Karlsruhe Institute of Technology (KIT), 76344 Eggenstein-Leopoldshafen, Germany; roman.popov@kit.edu (R.P.); varanuseremius@gmail.com (H.S.); 2 The National Natural History Collections, The Hebrew University of Jerusalem, Jerusalem 91904, Israel; 3 Laboratory AGEIS EA 7407, Team Tools for e-GnosisMedical & LabcomCNRS/UGA/OrangeLabs Telecoms4Health, Faculty of Medicine, Université Grenoble Alpes, F-38700 La Tronche, France

**Keywords:** standard genetic codes, codon assignment, tRNA, aminoacyl-tRNA synthetase classes

## Abstract

We propose combinatorial fusion rules that describe the codon assignment in the standard genetic code simply and uniformly for all canonical amino acids. These rules become obvious if the origin of the standard genetic code is considered as a result of a fusion of four protocodes: Two dominant AU and GC protocodes and two recessive AU and GC protocodes. The biochemical meaning of the fusion rules consists of retaining the complementarity between cognate codons of the small hydrophobic amino acids and large charged or polar amino acids within the protocodes. The proto tRNAs were assembled in form of two kissing hairpins with 9-base and 10-base loops in the case of dominant protocodes and two 9-base loops in the case of recessive protocodes. The fusion rules reveal the connection between the stop codons, the non-canonical amino acids, pyrrolysine and selenocysteine, and deviations in the translation of mitochondria. Using fusion rules, we predicted the existence of additional amino acids that are essential for the development of the standard genetic code. The validity of the proposed partition of the genetic code into dominant and recessive protocodes is considered referring to state-of-the-art hypotheses. The formation of two aminoacyl-tRNA synthetase classes is compatible with four-protocode partition.

## 1. Introduction

Covering more than 50 years of the literature on the origin of the genetic code, renowned specialists E.V. Koonin et al. put a shortlist of widespread statements about the code properties and aspects of its evolution [1]:

1. “The code is effectively universal: Departures from code universality in extant organisms are minor and of secondary origin.
2. The code is non-randomly organized and is highly robust to errors, although it is far from being globally optimal.
3. Evolution of the code involved expansion from a limited set of primordial amino acids toward the modern canonical set.”

We doubt that statement 3 from this list is a significant prerequisite for clarifying the codon assignment in the standard genetic code (SGC). The most commonly used arguments for the sequential entry of amino acids into SGC are differences in the prebiotic abundance of amino acids [2,3], indications of GC-rich content in the most archaic RNAs [4], or branching history of two aminoacyl-tRNA synthetase (aaRS) superfamilies [5]. Even though canonical amino acids and RNAs with a specific base content may have appeared on Earth in different ways and at different times, there is no evidence that the non-randomly organized SGC arose as a result of successive expansions with amino acids. R.D. Knight et al. critically reviewed hypotheses based on phylogenetic analysis and emphasized the absence of any evidence of code expansion during the evolution of synthetases [6].

Indeed, such a “progressively evolutionary” view is rather a common perception of historical processes. Maybe intuitive acceptance of statement 3 is the reason for criticisms (also by Koonin et al. [1]) of code origin hypotheses, such as the stereochemical hypothesis, that “failed to provide clear solutions” or “does not find general confirmation” [6,7]. Despite rapidly growing genomic databases, aptamer screening technology, and extensive computational efforts, the SGC origins remain unknown, even called the universal enigma [8].

The central issue of the genetic code origin is a rational explanation for the assignment of amino acids to different numbers of codons [9]. Several mathematical approaches of the genetic code in terms of symmetry properties and group theory have been developed [10,11,12]. The problem with such descriptions is the difficulty in providing a biological interpretation. The tessera hypothesis follows a different approach [13]. It is a unified mathematical framework that accounts for the degeneracy properties of both nuclear and mitochondrial genetic codes. According to this model, the early versions of the genetic code had codons of four base length. This assumption would solve the conundrum that regular triplet codon–anticodon duplexes are too unstable to allow primitive ribosome-free translation [14]. Indeed, predicted coding by quadruplet codons increases with mean body temperature in lizards [15] and coevolves with predicted tRNAs with expanded anticodons [16]. There is a row of observations originating from tRNAs with expanded eight-nucleotide anticodon loops [17,18,19] or mass spectrometry analyses of peptides corresponding to the translation of the human mitogenome according to codons with more than three nucleotides [20,21]. These observations could support the tessera hypothesis, but they are not necessarily evidencing a primitive genetic code with expanded codons. The tessera hypothesis proposes a rather indirect way to the SGC via transformation of the tessera code with codons of four nucleotides to the Juke’s ancestral code with codons of three nucleotides (16 amino acids and two stop codons) [22]. Using further assumptions, Juke postulated two additional successive expansions of the ancestral code (via Ile and Thr, and later via Met and Trp) to finally fully describe the SGC.

Instead of introducing four-base codons, we describe the exact degeneracy of the genetic code with simple fusion rules. In this study, we consider four single base-pair protocodes as originally independent coexisting codes.

## 2. Results

### 2.1. Single Base-Pair Codes and Combinatorial Rules of Their Fusion

Table 1 and Table 2 represent the standard genetic code but in a special form of four protocodes. Twenty proteinogenic amino acids are distributed over the two dominant and two recessive protocodes. The terms “dominant” and “recessive” are borrowed from classical genetics and refer to the fact that the dominant protocodes do not change their initial codon/amino acid assignments after the fusion. In contrast, the recessive codes acquire new triplets.

The peculiarity of this construction is that the number and type of codons for each amino acid in the SGC are determined according to rules 1–3. The red letters in Table 1 illustrate the changes according to these rules. The asterisks indicate the codon assignments of the SGC that we will discuss in the next sections.

Rule 1: The second-position bases do not change in any code.

Rule 2: A and G, as well as U and C, are exchangeable only in the third-position base in the dominant protocodes.

Rule 3: A and C, as well as U and G, are exchangeable either in the first position or simultaneously in the first and third positions in the recessive protocodes.

The derivation of these rules occurs automatically as soon as the coexistence of dominant and recessive codes is accepted. The advantage of these rules is that they are uniform for all amino acids and reduce the problem of codon assignment to a simple mathematical function.

Following the fusion rules, one can calculate the number of codons for the stop codon and each amino acid. For example, the stop codon UAG of the SGC originates from the stop codon UAA by substitution of A for G. Amino acid Lys has only two codons AAA and AAG in the SGC, because Lys had only one codon AAA in the protocode.

Each protocode contains only one positively charged amino acid (dominant codes—Lys and Arg and recessive codes—His and Arg (X4)). These positively charged amino acids may significantly contribute to the specific interactions between negatively charged RNAs and protopeptides.

### 2.2. Combinatorial Fusion Rules Preserve Complementarity of Codons for Specific Clusters of Amino Acids

Table 3 shows the four protocodes in form of complementary clusters **AUa**, **GCa**, **AUā,** and **GCā.** These clusters emerged automatically by writing down the complementary codons within the protocodes. It turned out that the properties of the amino acids significantly differ between complementary clusters. Clusters **AUā** and **GCā** include all hydrophobic canonical amino acids Met, Leu, Ile, Val, Ala, Pro, Phe, and two small amino acids Ser and Thr. In contrast, clusters **AUa** and **GCa** include only charged and polar amino acids. These four clusters represent the well-known evolutionary columns [7] that are frequently used to demonstrate the hypotheses of the genetic code expansion from the single Gly code to the SGC [23].

Table 4 shows the distribution of the complementary codons and corresponding amino acids after the fusion in the SGC. This complementarity has already been noticed by Rodin and Ohno [24]. The complementarity of the codons after the fusion changed in such a way that additional complementary codons appeared in the dominant protocodes. For example, a new pair Lys-Leu (AAG-CUU) is added to the Lys-Phe (AAA-UUU) from the protocode. The complementarity of amino acid codons in the recessive protocodes changed totally because the old coexisting codes disappeared. However, fusion rules preserved the original distribution of amino acids within the clusters **AUa**, **GCa**, **AUā,** and **GCā.**

## 3. Discussion

### 3.1. Kissing Proto tRNAs

The fact of codon complementarity before and after the fusion indicates the importance of the specific loop-loop interactions (kissing) between the proto tRNAs for the ancient translation. The kissing contacts were experimentally detected in the case of bacterial and viral systems, where they are prevalent in regulatory complexes [25,26]. This is also in line with the self-referential hypothesis for genetic code origins that assumes “kissing” between complementary tRNA anticodons [27,28], forming a structure similar to the ribosomal peptide elongation core [29]. The complementary hairpin kissing complexes are relatively stable. They demonstrate dissociation constants in the low-to-medium nanomolar range [25,30,31,32,33].

**Figure 1:**
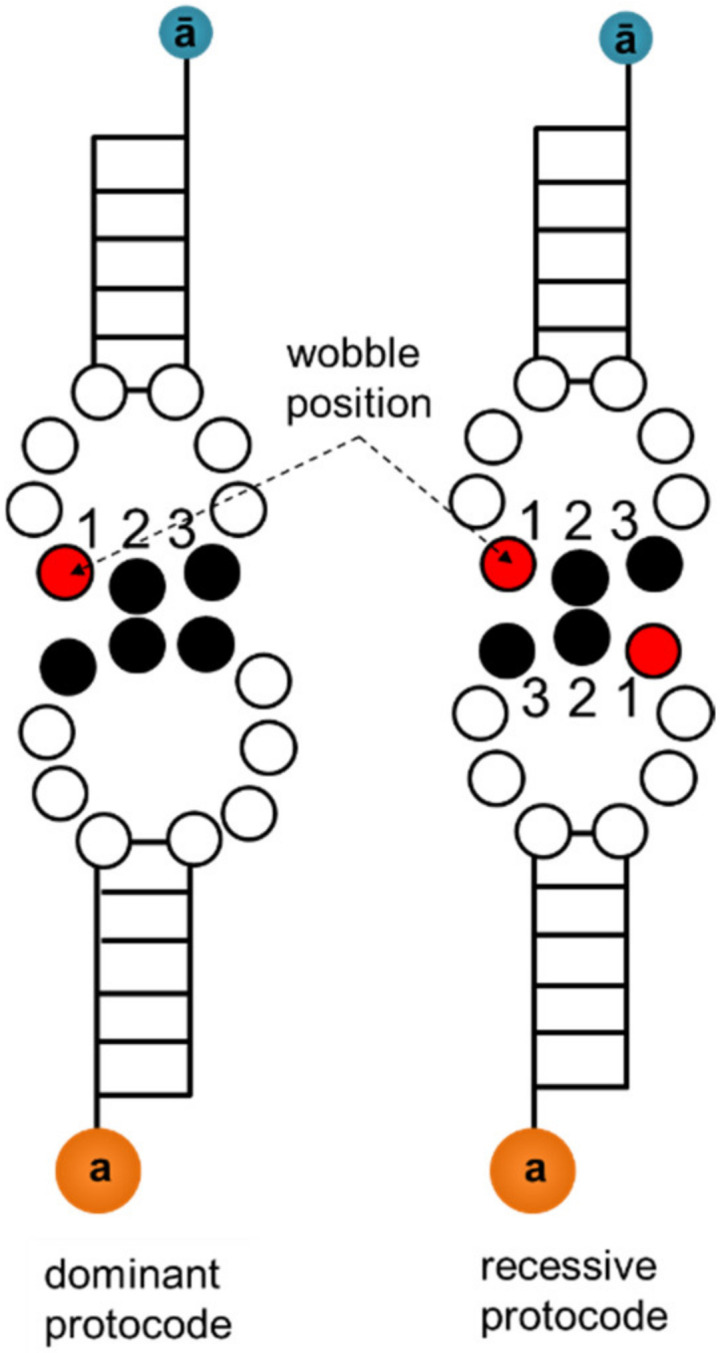
Schematic view of kissing proto tRNAs in form of hairpins. The red circle represents the wobble position. (**left**) Dominant protocode: kissing contact via a 9-base loop and a 10-base loop. The geometry of wobble positions corresponds to fusion rules 2. (**right**) Recessive protocode: kissing contact via two 9-base loops. The geometry of wobble positions corresponds to fusion rule 3.

Fusion rules represent the discrimination of A/G and U/C. This discrimination in codon recognition is known as a wobble position in the anticodon of the modern-type tRNA [23]. The wobble position is occupied by a modified base that is part of the universal genetic code and was probably present in Last Universal Common Ancestor (LUCA) [34]. Fusion rule 2 applies the A/G and U/C discrimination to the 3rd codon (1st anticodon) position. Thus, fusion rule 2 preserves the kissing contact between the 10-base loop of the proto tRNAs for the amino acids from the clusters **AUa** and **GCa** and the 9-base loop of the corresponding proto tRNAs for the amino acids from the clusters **AUā** and **GCā** (Figure 1, left).

The kissing contact in the recessive codes is represented by the two 9-base loops (Figure 1, right). This loop kissing allows for A/G and U/C discrimination both in the 1st and 3rd codon positions. Thus, fusion rule 3 has the same function as rule 2 to preserve the kissing contacts in the protocode after the fusion. The recessive protocodes lost their initial codon assignments after the fusion, because the new codons formed kissing loops with stronger affinity. For example, Gln-Leu tRNA kissing was initially formed by the complementary codons AAA-UUU (Table 3). After the fusion, the kissing Gln-Leu tRNA geometry was extended with codon pairs CAA-UUG und CAG-CUG (Table 4) with a greater affinity that made the initial assignment unnecessary.

The difference in the size of kissing loop geometries between the dominant and recessive protocodes caused the orthogonality of the ancient coexisting translation apparatuses.

The formation of proto tRNA pairs provided the advantage for their better recognition by the ancient aminoacyl-tRNA synthetases (proto-aaRS): (i) each pair had a more complex structure in comparison with a single hairpin; (ii) each pair was equipped with a small, mostly hydrophobic amino acid that caused a better affinity to the proto-aaRS.

The proto tRNAs with 9-base loop and 10-base loop hairpins give a clue about the emergence of the modern-type tRNA. D-loop and D-stem of the modern tRNA probably descended from 10-base loop proto tRNAs, and T-loop and T-stem from 9-base loop proto tRNAs.

### 3.2. Stop Codons, Noncanonical Amino Acids, and Deviations from SGC in Mitochondria

The combinatorial fusion rules establish a strong correlation between the stop codons, non-canonical amino acids, and deviation from the SGC in mitochondria. For example, stop codons UAG and UGA code the non-canonical amino acids Pyl and Sec. The list of the deviations from the SGC in the mitochondria [35] exactly matches the codon reassignments during the fusion (Table 5).

**Figure 2:**
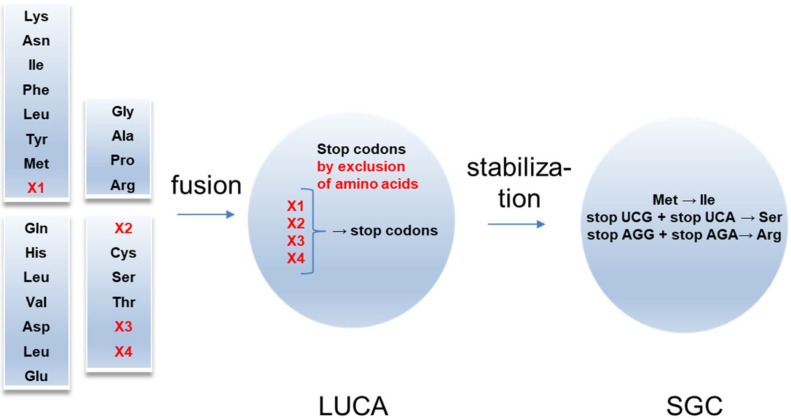
Origin of the SGC from the four-code fusion. Four primordial codes included four additional amino acids. After fusion, LUCA appeared. X1-X4 amino acids were excluded in favor of stop codons. In SGC, the part of stop codons was substituted by Ser and Arg.

These experimental results allow for the following evolutionary scenario of the SGC around the four-code fusion (Figure 2). The fusion might be considered as the origin of the LUCA. Initially, LUCA should additionally include X1–X4 amino acids. Their exclusion resulted in generating stop codons, which significantly reduced the stochastic translation of the amino acid sequences.

This conclusion correlates with the “ambush” hypothesis [36,37]. Along with this hypothesis, an adaptive mechanism mitigates the effects of slippage prone ribosomes by increasing the density of off-frame stop codons. Such a mechanism is reasonable to compensate for reduced translation efficiency in the case of unstable rRNAs. The loss of amino acid X1 from the dominant AU protocode resulted in the two stop codons UAA and UAG (fusion rule 2 for the dominant code). The stop codon UAG was adapted by prokaryotes for the non-canonical amino acid Pyl under evolutionary pressures to develop the methane metabolism [38,39]. The recessive GC code had lost the most amino acids after the fusion. Referring to the deviation from the SGC in mitochondria, Trp was very probably the amino acid X2. After the fusion, one of its triplets UGA became a stop codon. This free codon became available for Sec during evolution [40]. Although Sec is found in the three domains of life, it is not universal in all organisms [41]. The origin of X3 and X4 is unknown. Probably, the primordial amino acid X3 had properties similar to Ser. X4 was probably similar to the positively charged amino acid Arg. An X3-candidate can be one of the extraterrestrial serine derivatives (isoserine, homoserine, and β-homoserine) recently found in significant amounts in the Murchison meteorite [42]. The extremophilic prokaryotes are characterized by a significant content of AGC, AGU, AGG, or AGA codons. In particular, thermophiles and barophiles have high AGG content (X4), although dominant Arg codons are not used to increase the content of the protein stabilizing arginine [43].

The recessive GC code has lost the largest number of amino acids. The lack of hydrophobic amino acids in its cluster **GCā** probably caused their loss.

The reduction in the stop- and start-codons towards the SGC indicates the development of a less error-prone translation system. The stop codons of X3 and X4 were replaced by Ser and Arg, while Met by Ile. These changes lead to the maximal number of codons assigned to Arg and Ser and explain the exceptional odd number of codons for Ile, Met, and Trp (3, 1, 1 correspondingly). This is in line with observations that the evolution of the mitochondrial genetic codes seems best reconstructed when assuming the insertion of amino acids at stop codons [44].

### 3.3. Protocodes and Modern-Type Aminoacyl-tRNA Classes

**Figure 3:**
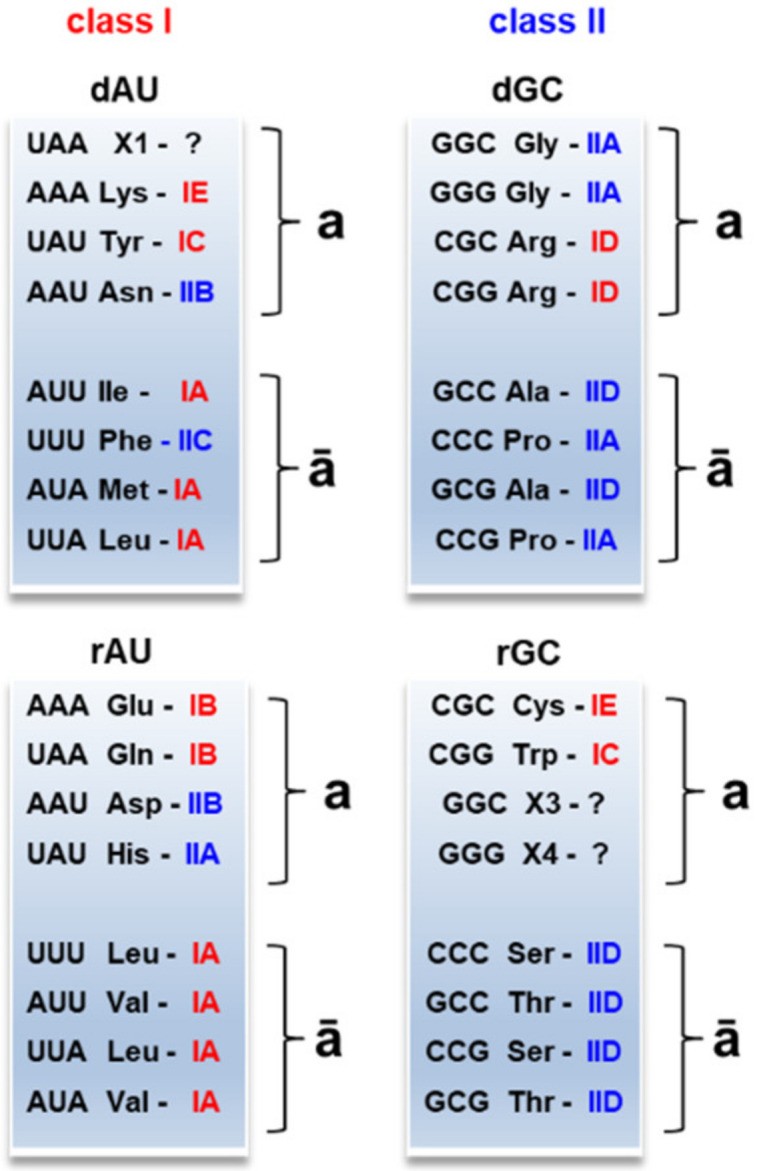
Distribution of the amino acids from the protocodes over two modern-type aminoacyl-tRNA classes and subclasses. The red color stands for aaRS class I, the blue for aaRS class II. The amino acids within clusters **ā** belong to the same aaRS class except for Phe. dAU and dGC indicate the dominant AU and GC protocodes, respectively. rAU and rGC indicate the recessive AU and GC protocodes.

The partition AU/GC affected the formation of the modern-type aaRSs (Figure 3). We use the definition of aaRS classes and subclasses and the corresponding amino acid assignments as presented in the review of Kim Y. et al. [45]. Amino acids from the recessive clusters **AUā** and **GCā** are catalyzed by the same aaRS subclasses IA and IID, respectively. Amino acids from the dominant clusters **AUā** and **GCā** show slight inhomogeneity in aaRS classes and subclasses. Charged and polar amino acids from the complementary clusters **a** exhibit significant inhomogeneity: IE, IC, IB, ID, IIB, and IIA.

Interestingly, the subsequent distribution of charged and polar amino acids over aaRS classes is associated with the initial codons from the protocodes. For example, Asn and Asp shared the same codon AAU in the protocodes, and both are catalyzed with the same aaRS subclass IIB. Arg and Cys shared the same codon CGC, and both are catalyzed with the same aaRS class I. By analogy, Arg and Trp (codon CGG) belong to the aaRS class I.

All amino acids from clusters **AUā** (Phe is an exception) belong to aaRS class I, and all amino acids from the **GCā** clusters belong to aaRS class II. Recall that these small, mostly hydrophobic amino acids may play a primary role in charging the proto-aaRS with amino acids from clusters **AUa** and **GCa**. Remarkable is the feature of the Phe-aaRS. Although Phe changed to aaRS class II, Phe retained its feature from the aaRS class I. Phe is coupled to the 2′OH of the ribose of the tRNA terminal adenosine [46]. In contrast, all aaRSs from class II attach amino acids to the 3′OH [47].

Note that some observations suggest that class I and class II tRNA synthetases originate from complementary strands of a single ancestral gene [48,49]. This gene would have originated from tRNA gene pairs coded by complementary strands of a given sequence [50].

### 3.4. Primordial Partition of the Genetic Code

As mentioned above, no assumptions about the evolutionary inclusion of the canonical amino acids into the genetic code are necessary to construct the SGC from the protocodes. Thus, the question about the evolution of the genetic code shifts to the question about the validity of the AU/GC partition. Is this just an unexpectedly simple mathematical trick or an indication of really coexisting ancient protocodes where two amino acids from different protocodes could share the same base triplet?

Besides the proposed AU/GC combinatorial partition, two additional nucleotide partitions exist: the purine/pyrimidine partition AG/CU (Table 6) and the keto/amino partition GU/AC (Table 7). Fusion rules specific to each of these partitions can be derived, to consider alternative fusion processes with exact mathematical descriptions of codon assignments. However, the alternative partitions AG/CU and GU/AC differ principally from the partition AU/GC. For AG/CU and GU/AC partitions, a significant number of new canonical amino acids should be assigned to the new codons after the fusion. This occurs in the case of amino acids presented in the SGC with two codons: Lys, Asn, Asp, Glu, Gln, His, and Phe. For example, two initial codons AAA and AAG of Lys (Table 7, first row) after fusion will be transformed to the codons AAU and AAC of Asn. Such fusions would require many additional assumptions that seem to be a significant disadvantage in comparison with the AU/GC partition. AU/GC partition includes most amino acids before the fusion.

### 3.5. Primordial Partition and Hypotheses on Amino Acid Inclusion Ranks in the Genetic Code

The partition AU/GC (as well the other AG/CU and AC/GU) imply the existence of the dominant and recessive protocodes. In this respect, we calculated the mean of the amino acid inclusion ranks in the genetic code for amino acids assigned to the dominant versus recessive protocodes assuming that one of the protocodes would be older than the other. Therefore, we used genetic code origin hypotheses from [52,53]. We also included in analyses some more recent, rather complete hypotheses, the self-referential model [27], and Rogers’s hypothesis [54]. Note that these hypotheses are congruent with the mean positions of amino acids in proteins [55,56] and with tRNA and ribosomal RNA secondary structures [57,58].

There was no difference between the mean genetic code inclusion ranks of amino acids coded by the dominant protocode pair vs. the remaining amino acids for fusion hypotheses based on the AU/GC and the AC/GU partitions. However, we found that amino acids coded by the dominant AG/CU protocodes are on average significantly more ancient than the remaining twelve amino acids, for most genetic code origin hypotheses, besides 11 among the 40 hypotheses reviewed by Trifonov [53]. The greatest congruence was with Harada and Fox experimental amino acid yields at high temperatures [59] with a statistical *p*-value of 6.4 × 10^−8^, followed by Miller’s experiment [60] with *p* = 3.3 × 10^−6^, and Wong’s nucleotide/amino acid metabolism coevolution hypothesis [61] with *p* = 4.3 × 10^−6^. Notable in this list are also hypotheses based on the amino acid contents of Murchison’s meteorite (*p* = 2.1 × 10^−5^) [62], the hypothesis by Rogers (*p* = 8.6 × 10^−5^) [54], the self-referential hypothesis (*p* = 0.0013), and the tRNA Urgen hypothesis of Eigen and Winkler-Oswatitsch (*p* = 0.0041) [63].

Thus, the averaging over the hypotheses, which are based on the step-by-step inclusion of amino acids into the code, do not identify any temporal relation between the dominant and recessive protocodes AU/GC.

The genetic code origin hypotheses reviewed by Trifonov [53] are not independent of each other and overall might have been selected for matching results of Miller’s experiment. In the next sections, we examine the relation of the primordial partition to other hypotheses that were not included in [53].

### 3.6. Primordial Partition and Self-Correcting Properties of the Natural Circular Code

The natural circular code consists of 20 codons that are overrepresented in the coding frame of genes as opposed to the remaining non-coding frames [64,65,66]. As a group, they have mathematical properties that enable the detection of the coding frame, a self-correcting property of genes, and of the genetic code. It is, hence, hypothesized that the natural circular code is somehow used by the ribosome to detect the coding frame. This assumption is strengthened by observations that specifically those ribosomal RNA regions that are in contact with mRNAs during translation are enriched in nucleotide triplets belonging to the natural circular code [67,68]. The natural circular code presumably arose as a result of selection for non-redundant coding in very short oligonucleotide chains [69,70].

The hypothesis that the natural circular code could have been an initial protocode is also strengthened by the observation that all amino acids coded by these 20 codons are listed as the most likely most ancient amino acids according to Miller’s experiment and related hypotheses. Hence, one would predict an overrepresentation of these circular code codons in at least one of the protocodes assumed by the fusion hypothesis. However, all these protocodes include exactly two codons belonging to the natural circular code, which is less than a third expected by chance. None of the dominant codes predicted by the fusion hypothesis converges with the natural circular code observed in natural genes and theoretical minimal RNA rings [71,72].

It is worth noting that the transition from the natural circular code to the SGC remains unexplored, while the transition from the coexisting protocodes to the SGC occurs automatically by the use of the universal and simple fusion rules. Very probably, the natural circular code was selected from the SGC in translation systems that are prone to frameshift errors under unstable environmental conditions. The natural circular code probably played a role in the genetic code evolution. However, the circular code does not explain the codon assignments in the SGC.

### 3.7. Primordial Partition and Ribosomal Structure

The three-dimensional structure of ribosomes may also include information about the genetic code and its origins. Nucleotide triplets in rRNA in direct contact with ribosomal proteins are biased in such a way that eight amino acids are selectively enriched near their respective codons and eleven amino acids are selectively enriched near their respective anticodons [73]. These observations suggest that anticodons and translation by tRNAs arose in a second phase of the evolution of the genetic code and the ribosome, while direct codon/amino acid contacts ruled the earliest translation mechanisms [51,74].

Thus, the fusion hypothesis would expect a distribution of amino acids within the protocodes according to these observations. For example, the earliest amino acids, coded by dominant protocodes, would have negative values if the bias for contacts with their codons is subtracted from the bias for contacts with their anticodons. The average of these differences was indeed negative for dominant AU/GC protocodes, and the average was positive for recessive AU/GC protocodes, but the difference was not statistically significant (one tailed *t* test, *p* = 0.147). No pattern was detected for the two remaining partition scenarios.

### 3.8. Primordial Partition and Codon/Amino Acid Affinities

The stereochemical hypothesis on genetic code origins derived from amino acid/nucleotide contacts in ribosomes is based on the stereochemical affinities between codons and amino acids [75,76,77]. This hypothesis is in line with affinities observed between mRNAs and the peptides they encode [78,79,80]. Observations indicate that triplet/amino acid affinities are highest for amino acids that presumably integrated earliest the genetic code. Presumed “more recent” amino acids have low affinities for their assigned nucleotide triplets [81]. We compare the affinities for the three primordial partition AU/GC, AG/CU, and the AC/GU, using the values as reported previously [51]. The only statistically relevant scenario was obtained for the AG/CU partition. In this case, dominant code assignments have greater codon/amino acid affinities than recessive code assignments in ten among thirteen cases (excluding stop codons, one-tailed sign test, *p* = 0.023, Table 6).

Hence, the dominant/recessive code division according to the AU/GC partition does not match the rationale of high/low affinities. However, the recent review on the stereochemical hypothesis taking into account high-throughput screens with aptamers leaves reasonable doubts that the weak specificity of amino acid interactions with RNA could play a central role in the code evolution [1].

### 3.9. How Could Protocodes Coexist?

AU/GC primordial partition distinguishes between only AU protocodes and only GC protocodes. However, chemical changes in A->G and G->A, as well as C->U and U->C are the most spontaneously occurring mutation types [82,83]. This implies that if one of the purines or one of the pyrimidines is available, the other purine, or the other pyrimidine, will spontaneously arise.

We believe that the four nucleotides and most of the canonical amino acids were available as building blocks before the formation of the protocodes and the SGC. The prerequisite of an existing protocode is the self-assembling of its building blocks to an ancient translation apparatus. Thus, if a building block does not involve interactions with such apparatus, its coexistence does not deliver the evidence that the protocode is not possible.

The protocode fusion can be divided into two stages. The first stage included the integration of G/C or A/U bases into the respective AU and GC protocodes. After this inclusion, the dominant and recessive protocodes could still exist as orthogonal translation systems, because new bases conserved the geometry of the kissing proto tRNAs. At this stage, the modern codon assignment of the most canonical amino acids was completed.

**Figure 4:**
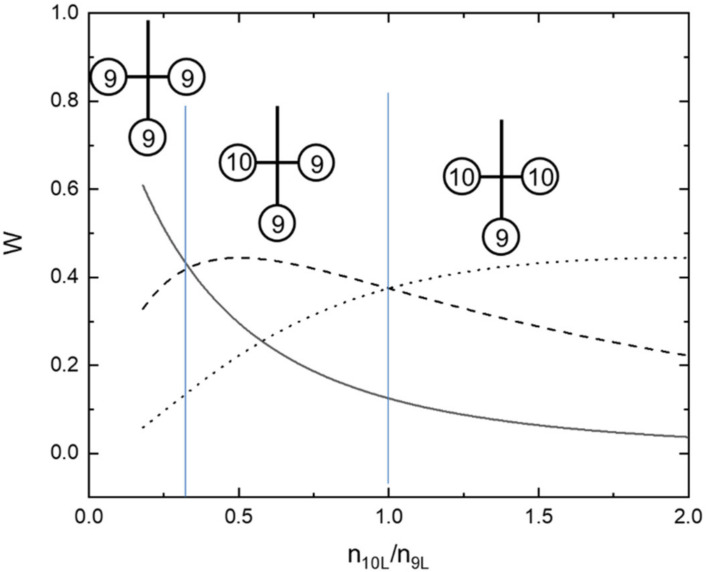
Probabilities W of three different 9-base and 10-base loop sets for the cloverleaf tRNA geometry versus the ratio of the 10-base loop concentration $n_{10L}\;$ to the 9-base loop concentration $n_{9L}$. The solid line corresponds to the loop set (9;9;9), the dashed line to (10;9;9), and the dotted line to (10;10;9). The probability of modern-type set (10;9;9) is the largest by $n_{10L}/n_{9L}$ in an interval (0.3:1).

In the second stage, modern tRNAs and the aaRS classes emerged. According to the different hypotheses, the modern tRNA was formed by a fusion of two [84,85] or three hairpins [86,87]. Assuming the random nature of this fusion and the equal number of complementary proto tRNAs in the respective protocodes, we evaluated the relationship between the proto tRNA concentrations in dominant and recessive codes. Figure 4 shows the probabilities of the loop sets within the cloverleaf geometry versus the ratio of the 10-base loop concentration to the 9-base loop concentration ν = $n_{10L}/n_{9L}$. As the cloverleaf has three positions and only two types of loops (9-base- and 10-base loop), these probabilities are described with known combinatorial formulas (Section 4.3).

The maximum of the probability for the modern type cloverleaf (10;9;9–D-loop, anticodon loop, T- loop) is achieved by ν = 0.5. As the ratio of the 10-base loop to 9-base loop hairpins in the protocodes was 1:3 (see Section 3.1), this value means that the concentration of proto tRNAs in the dominant codes was twice as high as in the recessive ones. If tRNA was formed according to the two-hairpin-fusion models, then this ratio should be even higher.

This estimation delivers a simple explanation for the orthogonality in translation between the dominant and recessive protocodes. The hairpins from the dominant codes just inhibited the recessive translation via specific binding to the complementary recessive hairpins according to fusion rule 2. Thus, the translation of the recessive protocodes could work only with leftover codons according to fusion rule 3.

The conclusion in Figure 4 can be used to support the statement that the D-loop of real tRNAs originated from one of the 10-base loop hairpins from the dominant protocodes that was outnumbered inside coexisting protocodes.

## 4. Materials and Methods

### 4.1. Amino Acid Inclusion Ranks, Chou-Fasman Conformational Indices, Protocodes, and Aminoacyl-tRNA Classes

Amino acid inclusion ranks in the genetic code were as reviewed by Trifonov [53]. Additional hypotheses were also considered by Guimarães et al. [28] and Rogers et al. [54]. Chou-Fasman conformational indices and other amino acid properties are from ProtScale [78,88].

### 4.2. Trinucleotide/Amino Acid Affinities

We used the calculated affinities of all 64 trinucleotides with all 20 amino acids summing single-nucleotide affinities for amino acids from [51]. Single nucleotide/amino acid affinity scores were calculated based on contact frequencies between nucleotides and amino acids in crystal structures of interacting RNA–protein complexes [88].

We did not use affinities in solution, only affinities as determined for surfaces. Affinities follow the Gibbs equation Δ*G*  =  −*RT*lnK_d_, with *R* is the gas constant, *T* temperature, and K_d_ the binding constant [88].

Polyansky and Zagrovic [41] estimated affinities as the negative of the log-transformed ratio between all observed contacts between an amino acid and a nucleotide in its assigned codons/anticodons (N^ij^_obs_) and the expected contact number assuming random contacts (N^ij^_exp_). Expected random contact frequencies are the product of the frequency of that amino acid in the protein(s) forming a complex with that RNA and the frequencies of nucleotides in that amino acid’s cognate codons/anticodons in that RNA:

ε^ij^ = −ln (N^ij^_obs_/N^ij^_exp_), (1)

where i = 1,…,20 for amino acids, and j = 1,…,4 for nucleotides. This estimates biases for these contacts in the 3D structure of the RNA–protein complex. This bias corresponds to the binding constant Kd in the Gibbs formulation of affinities: the binding constant is proportional to the bias for observed vs. expected contacts. Note that these ratios are dimensionless and have no unit. Standard quasi-chemical approximations estimate amino acid/amino acid contact energies to predict protein structures and their stabilities [89,90]. The same principles are applied in the context of nucleotide/amino acid contacts.

### 4.3. Probability of the Emergence of Different Loop Sets for the Cloverleaf Geometry as a Result of a Random Fusion of 9-Base Loop and 10-Base Loop Hairpins

We assume that

(2) $$\nu \;=\;n_{10L}/n_{9L},$$

where $n_{10L}$ is the concentration of the hairpins with the 10-base loop, and $n_{9L}$ the concentration of the hairpins with the 10-base loop. This implies that the probabilities of the emergence of the cloverleaf sets: Three 9-base loops W(9,9,9), two 9-base loops with one 10-base loop W(10,9,9), and two 10-base loops with one 9-base loop W(10,10,9):

(3) $$W(9,9,9)\;=\;{\left(\frac{1}{1+\nu }\right)}^{3},$$

(4) $$W(10,9,9)\;=\;3\frac{\nu }{1+\nu }{\left(\frac{1}{1+\nu }\right)}^{2},$$

(5) $$W(10,10,9)\;=\;3{\left(\frac{\nu }{1+\nu }\right)}^{2}\frac{1}{1+\nu }$$

Here, W(9,9,9), W(10,9,9), and W(10,10,9) are probabilities of the emergence of the cloverleaf sets: Three 9-base loops, two 9-base loops with one 10-base loop, and two 10-base loops with one 9-base loop, respectively.

## 5. Conclusions

The fusion rules are no hypothesis, but the mathematical reality of the SGC. According to our knowledge, the AU/GC partition and the fusion rules are the simplest analytical way to describe the emergence of the SGC. Why has this solution not been noticed for more than half a century since the discovery of the code table? Firstly, the fusion rules contradict the postulates of the gradual expansion of the genetic code. This postulate has long dominated the science of the origin of the genetic code. Its cognitive potential is currently being questioned. Secondly, fusion rules imply coexisting protocodes. Interest in orthogonal translation systems has grown only in recent years. The question of why evolution did not use the same orthogonal approach seems no longer to be abstract.

Using the fusion rules, we propose a fusion hypothesis of the origin of the genetic code. The fusion hypothesis states that the SGC originated from the four real protocodes. Their biochemical meaning consists of retaining the complementarity of the codons of the “small” amino acids from the clusters **ā** to the codons of the “large“ amino acids from the clusters **a**. Before the fusion, most of the canonical amino acids were already involved in the coexisting translational apparatuses of the protocodes. Our hypothesis proposes the existence of kissing proto tRNAs responsible for the emergence of the SGC code. The combinatorial fusion rules established the connections between the stop codons, non-canonical amino acids, and the deviation from the standard genetic codes in mitochondria.

Two alternative partitions of the genetic code AG/CU and AC/GU were also examined. The AC/GU partition would reflect keto-amino groupings of nucleotides, which imply relatively rare isoforms of nucleotides. The AG/CU partition reflects a purine/pyrimidine grouping of nucleotides. This partition, unlike the two other partitions, is statistically compatible with most historical hypotheses of integration ranks of amino acids in the genetic code. However, both alternative partitions require additional assumptions for expanding genetic codes after the fusion.

The large diversity of code origin hypotheses including the stereochemical hypothesis produce partially congruent predictions about the historical integration of amino acids into the code. This means that the genetic code, as we know, is compatible with a large number of evolutionary scenarios. Hence, the healthiest approach to this problem is no “natural selection” between hypotheses, because they probably reflect more or less different independent periods/conditions of the code’s development. In contrast to most hypotheses, the fusion hypothesis exactly generates the SGC at its last stage.

The fusion hypothesis raises new questions: How did the protocodes appear? What amino acids are missing after the protocode fusion? How was the transition from protopeptide-synthetases to the modern-type aaRSs? Answering these questions requires experimental research. Many powerful methods are available today for screening peptide interactions with various targets, including phage display and peptide arrays. From the experimental point of view, the fusion hypothesis has an advantage. It allows for the study of primordial translation mechanisms with a reduced number of amino acids within single protocodes.

## Figures and Tables

**Table 1:** Dominant AU and GC protocodes and their transformation to the standard genetic code (SGC) after the fusion. The codons of the SGC are obtained due to mutations A↔G or U↔C in the third position of the protocodes. The red letters illustrate the transformation.

AU Code			GC Code		
Amino Acid	Before Fusion	SGC	Amino Acid	Before Fusion	SGC
Lys	AAA	AAA, AAG	Gly	GGG	GGG, GGA
Asn	AAU	AAU, AAC	Gly	GGC	GGC, GGU
Ile	AUU	AUU, AUC	Ala	GCC	GCC, GCU
Phe	UUU	UUU, UUC	Ala	GCG	GCG, GCA
Leu	UUA	UUA, UUG	Pro	CCC	CCC, CCU
Tyr	UAU	UAU, UAC	Pro	CCG	CCG, CCA
Met (Ile + Met) *	AUA	AUA, AUG	Arg	CGC	CGC, CGU
stop	UAA	UAA, UAG	Arg	CGG	CGG, CGA

* *The asterisk indicates the codon assignments of the SGC that we will discuss in the next sections.*

**Table 2:** Recessive AU and GC protocodes and their transformation to the SGC after the fusion. The codons of the SGC are obtained due to mutations A↔G or U↔C in the first position or the first and the third positions of the protocodes. The red letters illustrate the transformation.

AU Code			GC Code		
Amino Acid	before Fusion	SGC	Amino Acid	before Fusion	SGC
Gln	UAA	CAA, CAG	stop (Trp + stop) *	CGG	UGG, UGA
His	UAU	CAU, CAC	Cys	CGC	UGC, UGU
Leu	UUU	CUU, CUC	Ser	CCC	UCC, UCU
Val	AUU	GUU, GUC	Ser	CCG	UCG, UCA
Val	AUA	GUA, GUG	Thr	GCC	ACC, ACU
Asp	AAU	GAU, GAC	Thr	GCG	ACG, ACA
Leu	UUA	CUA, CUG	Ser *	GGC	AGC, AGU
Glu	AAA	GAA, GAG	Arg *	GGG	AGG, AGA

* *The asterisks indicate the codon assignments of the SGC that we will discuss in the next sections.*

**Table 3:** Distribution of the amino acids within the protocodes into the complementary clusters **AUa**, **GCa**, **AUā,** and **GCā**. Clusters **AUā** and **GCā** consist of only the “small” amino acids. **AUa** and **GCa** consist only of the charged or polar amino acids.

Amino Acids, Cluster a	AU Codons, Cluster a	AU Codons, Cluster ā	Amino Acids, Cluster ā	Amino Acids, Cluster a	GC Codons, Cluster a	GC Codons, Cluster ā	Amino Acids, Cluster ā
Lys	AAA	UUU	Phe	Gly	GGG	CCC	Pro
Asn	AAU	AUU	Ile	Gly	GGC	GCC	Ala
Tyr	UAU	AUA	Ile (Ile+ Met) *	Arg	CGC	GCG	Ala
stop	UAA	UUA	Leu	Arg	CGG	CCG	Pro
Gln	UAA	UUA	Leu	Trp + stop	CGG	CCG	Ser
His	UAU	AUA	Val	Cys	CGC	GCG	Thr
Asp	AAU	AUU	Val	Ser *	GGC	GCC	Thr
Glu	AAA	UUU	Leu	Arg *	GGG	CCC	Ser

* *The asterisks indicate the codon assignments of the SGC that we will discuss in the next sections.*

**Table 4:** Distribution of the amino acids within the SGC into the complementary clusters **AUa**, **GCa**, **AUā,** and **GCā**. The distribution into the clusters remains after the fusion.

Amino Acids, Cluster a	AU Codons, Cluster a	AU Codons, Cluster ā	Amino Acids, Cluster ā	Amino Acids, Cluster a	GC Codons, Cluster a	GC Codons, Cluster ā	Amino Acids, Cluster ā
Lys Lys	AAA AAG	UUU CUU	Phe Leu	Gly Gly	GGG GGA	CCC UCC	Pro Ser
Asn Asn	AAU AAC	AUU GUU	Ile Val	Gly Gly	GGC GGU	GCC ACC	Ala Thr
Tyr Tyr	UAU UAC	AUA GUA	Met Val	Arg Arg	CGC CGU	GCG ACG	Ala Thr
stop stop	UAA UAG	UUA CUA	Leu Leu	Arg Arg	CGG CGA	CCG UCG	Pro Ser
Gln Gln	CAA CAG	UUG CUG	Leu Leu	Trp stop	UGG UGA	CCA UCA	Pro Ser
His His	CAU CAC	AUG GUG	Met Val	Cys Cys	UGC UGU	GCA ACA	Ala Thr
Asp Asp	GAU GAC	AUC GUC	Ile Val	Ser * Ser *	AGC AGU	GCU ACU	Ala Thr
Glu Glu	GAA GAG	UUC CUC	Phe Leu	Arg * Arg *	AGG AGA	CCU UCU	Pro Ser

* *The asterisks indicate the codon assignments of the SGC that we will discuss in the next sections.*

**Table 5:** Deviations from the SGC in mitochondria and the protocode fusion involving start and stop codons.

Occurrence	Codon	SGC	Deviation	Protocode Fusion
Mitochondria by all studied organisms	UGA	stop	Trp	stop GGG → Trp UGG + stop UGA (fusion rule 3)
Vertebrate mitochondria, *Drosophila,* and protozoa	AUA	Ile	Met	Ile AUA → Ile AUA + Met AUG (fusion rule 2)
Invertebrate mitochondria	AGG, AGA	Arg	Ser	Arg GGG → Ser AGC + Ser AGU (fusion rule 3)
Vertebrate mitochondria	AGG AGA,	Arg	stop	Arg GGG → Arg AGG + Arg AGA (fusion rule 3)
*Drosophila*	AGA	Arg	stop	Arg GGG → Arg AGG + Arg AGA (fusion rule 3)

**Table 6:** AG/CU partition before the fusion. Codons are followed by their codon/amino acid affinity [51] according to the dominant/recessive protocodes. The affinities are dimensionless (Section 3.8 and Section 4.2).

AG Dominant			AG Recessive		CU Dominant			CU Recessive	
Shared Codon	Amino Acid	Affinity	Amino Acid	Affinity	Shared Codon	Amino Acid	Affinity	Amino Acid	Affinity
AAA	Lys	−27	Stop	n.d.	CCC	Pro	−27	Ala	−51
AAG	Lys	−7	Stop	n.d.	CCU	Pro	6	Ala	−1
AGA	Arg	−7	Trp	−17	CUC	Leu	−19	Val	−1
AGG	Arg	4	Stop	n.d.	CUU	Leu	7	Val	22
GAA	Glu	−54	Arg	−18	UCC	Ser	−51	Ile	−24
GAG	Glu	−23	Gln	29	UCU	Ser	−40	Met	−18
GGA	Gly	−20	Arg	−7	UUC	Phe	−40	Thr	−36
GGG	Gly	−55	Gln	1	UUU	Phe	28	Thr	36

**Table 7:** AC/GU partition before the fusion. Codons are followed by their codon/amino acid affinity according to the dominant/recessive protocodes. The affinities are dimensionless (Section 3.8 and Section 4.2).

AC Dominant			AC Recessive		GU Dominant			GU Recessive	
Shared Codon	Amino Acid	Affinity	Amino Acid	Affinity	Shared Codon	Amino Acid	Affinity	Amino Acid	Affinity
AAA	Lys	−27	Asp	−18	GGG	Gly	−55	Ser	−51
AAC	Asn	86	Glu	52	GGU	Gly	−24	Arg	−4
ACA	Thr	−68	Ala	−37	GUG	Val	14	Ile	26
ACC	Thr	−88	Ala	−44	GUU	Val	−5	Ile	2
CAA	Gln	35	Ser	−50	UGG	Trp	27	Arg	−4
CAC	His	13	Ser	−79	UGU	Cys	−1	Arg	−4
CCA	Pro	−37	Tyr	6	UUG	Leu	−12	Leu	−12
CCC	Pro	−27	Stop	n.d.	UUU	Phe	28	Leu	−47

## Abbreviations

SGC	standard genetic code
aaRS	aminoacyl-tRNA synthetase
tRNA	transfer RNA
stop	stop codon
Lys	lysine
Asn	asparagine
Asp	aspartic acid
Ile	isoleucine
Phe	phenylalanine
Leu	leucine
Tyr	tyrosine
Met	methionine
Pyl	pyrrolysine
Sec	selenocysteine
Gly	glycine
Ser	serine
Ala	alanine
Pro	proline
His	histidine
Val	valine
Arg	arginine
Cys	cysteine
Gln	glutamine

## References

[1] Koonin E.V., Novozhilov A.S. (2017). Origin and Evolution of the Universal Genetic Code. Annu. Rev. Genet..

[2] Chatterjee S., Yadav S. (2019). The Origin of Prebiotic Information System in the Peptide/RNA World: A Simulation Model of the Evolution of Translation and the Genetic Code. Life.

[3] Higgs P.G., Pudritz R.E. (2009). A Thermodynamic Basis for Prebiotic Amino Acid Synthesis and the Nature of the First Genetic Code. Astrobiology.

[4] Gospodinov A., Kunnev D. (2020). Universal Codons with Enrichment from GC to AU Nucleotide Composition Reveal a Chronological Assignment from Early to Late Along with LUCA Formation. Life.

[5] Shore J.A., Holland B.R., Sumner J.G., Nieselt K., Wills P.R. (2020). The Ancient Operational Code is Embedded in the Amino Acid Substitution Matrix and aaRS Phylogenies. J. Mol. Evol..

[6] Knight R.D., Freeland S.J., Landweber L.F. (1999). Selection, history and chemistry: The three faces of the genetic code. Trends Biochem. Sci..

[7] Higgs P.G. (2009). A four-column theory for the origin of the genetic code: Tracing the evolutionary pathways that gave rise to an optimized code. Biol. Direct.

[8] Koonin E.V., Novozhilov A.S. (2009). Origin and Evolution of the Genetic Code: The Universal Enigma. IUBMB Life.

[9] Philip G.K., Freeland S.J. (2011). Did Evolution Select a Nonrandom "Alphabet" of Amino Acids?. Astrobiology.

[10] Antoneli F., Forger M. (2011). Symmetry breaking in the genetic code: Finite groups. Math. Comput. Model..

[11] Hornos J.E.M., Hornos Y.M.M. (1993). Algebraic Model for the Evolution of the Genetic-Code. Phys. Rev. Lett..

[12] Lenstra R. (2014). Evolution of the genetic code through progressive symmetry breaking. J. Theor. Biol..

[13] Gonzalez D.L., Giannerini S., Rosa R. (2019). On the origin of degeneracy in the genetic code. Interface Focus.

[14] Baranov P.V., Venin M., Provan G. (2009). Codon Size Reduction as the Origin of the Triplet Genetic Code. PLoS ONE.

[15] Seligmann H., Labra A. (2013). Tetracoding increases with body temperature in Lepidosauria. Biosystems.

[16] Seligmann H. (2014). Putative anticodons in mitochondrial tRNA sidearm loops: Pocketknife tRNAs?. J. Theor. Biol..

[17] Riddle D.L., Carbon J. (1973). Frameshift suppression: A nucleotide addition in the anticodon of a glycine transfer RNA. Nat. New Biol..

[18] Atkins J.F., Bjork G.R. (2009). A gripping tale of ribosomal frameshifting: Extragenic suppressors of frameshift mutations spotlight P-site realignment. Microbiol. Mol. Biol. Rev..

[19] Atkins J.F. (2018). Culmination of a half-century quest reveals insight into mutant tRNA-mediated frameshifting after tRNA departure from the decoding site. Proc. Natl. Acad. Sci. USA.

[20] Seligmann H. (2017). Natural mitochondrial proteolysis confirms transcription systematically exchanging/deleting nucleotides, peptides coded by expanded codons. J. Theor. Biol..

[21] Seligmann H. (2015). Codon expansion and systematic transcriptional deletions produce tetra-, pentacoded mitochondrial peptides. J. Theor. Biol..

[22] Jukes T.H. (1973). Possibilities for the evolution of the genetic code from a preceding form. Nature.

[23] Lei L., Burton Z.F. (2020). Evolution of Life on Earth: tRNA, Aminoacyl-tRNA Synthetases and the Genetic Code. Life.

[24] Rodin S.N., Ohno S. (1997). Four primordial modes of tRNA-synthetase recognition, determined by the (G,C) operational code. Proc. Natl. Acad. Sci. USA.

[25] Salim N., Lamichhane R., Zhao R., Banerjee T., Philip J., Rueda D., Feig A.L. (2012). Thermodynamic and Kinetic Analysis of an RNA Kissing Interaction and Its Resolution into an Extended Duplex. Biophys. J..

[26] Paillart J.C., Skripkin E., Ehresmann B., Ehresmann C., Marquet R. (1996). A loop-loop "kissing’’ complex is the essential part of the dimer linkage of genomic HIV-1 RNA. Proc. Natl. Acad. Sci. USA.

[27] Guimaraes R.C. (2017). Self-Referential Encoding on Modules of Anticodon Pairs-Roots of the Biological Flow System. Life.

[28] Guimaraes R.C., Moreira C.H.C., de Farias S.T. (2008). A self-referential model for the formation of the genetic code. Theor. Biosci..

[29] Agmon I. (2018). Hypothesis: Spontaneous Advent of the Prebiotic Translation System via the Accumulation of L-Shaped RNA Elements. Int. J. Mol. Sci..

[30] Durand G., Dausse E., Goux E., Fiore E., Peyrin E., Ravelet C., Toulme J.J. (2016). A combinatorial approach to the repertoire of RNA kissing motifs; towards multiplex detection by switching hairpin aptamers. Nucleic Acids Res..

[31] Windbichler N., Werner M., Schroeder R. (2003). Kissing complex-mediated dimerisation of HIV-1 RNA: Coupling extended duplex formation to ribozyme cleavage. Nucleic Acids Res..

[32] Wallace M.I., Ying L.M., Balasubramanian S., Klenerman D. (2001). Non-Arrhenius kinetics for the loop closure of a DNA hairpin. Proc. Natl. Acad. Sci. USA.

[33] Kushiro T., Schimmel P. (2002). Trbp111 selectively binds a noncovalently assembled tRNA-like structure. Proc. Natl. Acad. Sci. USA.

[34] Weiss M.C., Preiner M., Xavier J.C., Zimorski V., Martin W.F. (2018). The last universal common ancestor between ancient Earth chemistry and the onset of genetics. PLoS Genet..

[35] Jukes T.H., Osawa S. (1990). The Genetic-Code in Mitochondria and Chloroplasts. Experientia.

[36] Seligmann H., Pollock D.D. (2004). The ambush hypothesis: Hidden stop codons prevent off-frame gene reading. DNA Cell Biol..

[37] Seligmann H. (2019). Localized Context-Dependent Effects of the "Ambush" Hypothesis: More Off-Frame Stop Codons Downstream of Shifty Codons. DNA Cell Biol..

[38] Srinivasan G., James C.M., Krzycki J.A. (2002). Pyrrolysine encoded by UAG in Archaea: Charging of a UAG-decoding specialized tRNA. Science.

[39] Hao B., Gong W.M., Ferguson T.K., James C.M., Krzycki J.A., Chan M.K. (2002). A new UAG-encoded residue in the structure of a methanogen methyltransferase. Science.

[40] Donovan J., Copeland P.R. (2010). The Efficiency of Selenocysteine Incorporation Is Regulated by Translation Initiation Factors. J. Mol. Biol..

[41] Longtin R. (2004). A forgotten debate: Is selenocysteine the 21st amino acid?. J. Natl. Cancer Inst..

[42] Koga T., Naraoka H. (2017). A new family of extraterrestrial amino acids in the Murchison meteorite. Sci. Rep..

[43] Khan M.F., Patra S. (2018). Deciphering the rationale behind specific codon usage pattern in extremophiles. Sci. Rep..

[44] Seligmann H. (2015). Phylogeny of genetic codes and punctuation codes within genetic codes. Biosystems.

[45] Kim Y., Opron K., Burton Z.F. (2019). A tRNA- and Anticodon-Centric View of the Evolution of Aminoacyl-tRNA Synthetases, tRNAomes, and the Genetic Code. Life.

[46] Sprinzl M., Cramer F. (1975). Site of Aminoacylation of Transfer-Rnas from Escherichia-Coli with Respect to 2’-Hydroxyl Group or 3’-Hydroxyl Group of Terminal Adenosine. Proc. Natl. Acad. Sci. USA.

[47] Moras D. (1992). Structural and Functional-Relationships between Aminoacyl-Transfer Rna-Synthetases. Trends Biochem. Sci..

[48] Martinez-Rodriguez L., Erdogan O., Jimenez-Rodriguez M., Gonzalez-Rivera K., Williams T., Li L., Weinreb V., Collier M., Chandrasekaran S.N., Ambroggio X. (2015). Functional Class I and II Amino Acid-activating Enzymes Can Be Coded by Opposite Strands of the Same Gene. J. Biol. Chem..

[49] Carter C.W., Li L., Weinreb V., Collier M., Gonzalez-Rivera K., Jimenez-Rodriguez M., Erdogan O., Kuhlman B., Ambroggio X., Williams T. (2014). The Rodin-Ohno hypothesis that two enzyme superfamilies descended from one ancestral gene: An unlikely scenario for the origins of translation that will not be dismissed. Biol. Direct.

[50] Rodin S.N., Rodin A.S. (2008). On the origin of the genetic code: Signatures of its primordial complementarity in tRNAs and aminoacyl-tRNA synthetases. Heredity.

[51] Seligmann H. (2020). First arrived, first served: Competition between codons for codon-amino acid stereochemical interactions determined early genetic code assignments. Sci. Nat.-Heidelberg.

[52] Demongeot J., Seligmann H. (2020). RNA Rings Strengthen Hairpin Accretion Hypotheses for tRNA Evolution: A Reply to Commentaries by ZF Burton and M. Di Giulio. J. Mol. Evol..

[53] Trifonov E.N. (2000). Consensus temporal order of amino acids and evolution of the triplet code. Gene.

[54] Rogers S.O. (2019). Evolution of the genetic code based on conservative changes of codons, amino acids, and aminoacyl tRNA synthetases. J. Theor. Biol..

[55] Seligmann H. (2018). Protein Sequences Recapitulate Genetic Code Evolution. Comput. Struct. Biotechnol. J..

[56] Demongeot J., Seligmann H. (2019). Theoretical minimal RNA rings recapitulate the order of the genetic code’s codon-amino acid assignments. J. Theor. Biol..

[57] Demongeot J., Seligmann H. (2020). Accretion history of large ribosomal subunits deduced from theoretical minimal RNA rings is congruent with histories derived from phylogenetic and structural methods. Gene.

[58] Demongeot J., Seligmann H. (2019). The Uroboros Theory of Life’s Origin: 22-Nucleotide Theoretical Minimal RNA Rings Reflect Evolution of Genetic Code and tRNA-rRNA Translation Machineries. Acta Biotheor..

[59] Fox S.W., Harada K. (1960). The Thermal Copolymerization of Amino Acids Common to Protein. J. Am. Chem. Soc..

[60] Miller S.L. (1953). A Production of Amino Acids under Possible Primitive Earth Conditions. Science.

[61] Wong J.T. (1975). A co-evolution theory of the genetic code. Proc. Natl. Acad. Sci. USA.

[62] Kvenvolden K., Lawless J., Pering K., Peterson E., Flores J., Ponnamperuma C., Kaplan I.R., Moore C. (1970). Evidence for extraterrestrial amino-acids and hydrocarbons in the Murchison meteorite. Nature.

[63] Eigen M., Winkler-Oswatitsch R. (1981). Transfer-RNA, an early gene?. Naturwissenschaften.

[64] Michel C.J. (2017). The Maximal C(3) Self-Complementary Trinucleotide Circular Code X in Genes of Bacteria, Archaea, Eukaryotes, Plasmids and Viruses. Life.

[65] Dila G., Michel C.J., Thompson J.D. (2020). Optimality of circular codes versus the genetic code after frameshift errors. Biosystems.

[66] Michel C.J. (2020). The maximality of circular codes in genes statistically verified. Biosystems.

[67] Dila G., Ripp R., Mayer C., Poch O., Michel C.J., Thompson J.D. (2019). Circular code motifs in the ribosome: A missing link in the evolution of translation?. RNA.

[68] Michel C.J., Thompson J.D. (2020). Identification of a circular code periodicity in the bacterial ribosome: Origin of codon periodicity in genes?. RNA Biol..

[69] Demongeot J., Seligmann H. (2019). Spontaneous evolution of circular codes in theoretical minimal RNA rings. Gene.

[70] Demongeot J., Seligmann H. (2020). Pentamers with Non-redundant Frames: Bias for Natural Circular Code Codons. J. Mol. Evol..

[71] Demongeot J., Besson J. (1996). The genetic code and cyclic codes. C. R. Acad. Sci. III.

[72] Demongeot J., Seligmann H. (2020). Why Is AUG the Start Codon? Theoretical Minimal RNA Rings: Maximizing Coded Information Biases 1st Codon for the Universal Initiation Codon AUG. Bioessays.

[73] Johnson D.B., Wang L. (2010). Imprints of the genetic code in the ribosome. Proc. Natl. Acad. Sci. USA.

[74] Demongeot J., Seligmann H. (2020). Theoretical minimal RNA rings mimick molecular evolution before tRNA-mediated translation: Codon-amino acid affinities increase from early to late RNA rings. C. R. Biol..

[75] Pelc S.R., Welton M.G.E. (1966). Stereochemical Relationship between Coding Triplets and Amino-Acids. Nature.

[76] Yarus M. (2017). The Genetic Code and RNA-Amino Acid Affinities. Life.

[77] Koonin E.V. (2017). Frozen Accident Pushing 50: Stereochemistry, Expansion, and Chance in the Evolution of the Genetic Code. Life.

[78] Polyansky A.A., Zagrovic B. (2013). Evidence of direct complementary interactions between messenger RNAs and their cognate proteins. Nucleic Acids Res..

[79] Bartonek L., Zagrovic B. (2017). mRNA/protein sequence complementarity and its determinants: The impact of affinity scales. PLoS Comput. Biol..

[80] Bartonek L., Braun D., Zagrovic B. (2020). Frameshifting preserves key physicochemical properties of proteins. Proc. Natl. Acad. Sci. USA.

[81] Kahana A., Lancet D. (2019). Protobiotic Systems Chemistry Analyzed by Molecular Dynamics. Life.

[82] Graur D., Li W.-H., Gojobori T. (1982). Patterns of nucleotide substitution in pseudogenes and funcftional genes. J. Mol. Evol..

[83] Francino M.P., Ochman H. (2001). Deamination as the basis of strand-asymmetric evolution in transcribed Escherichia coli sequences. Mol. Biol. Evol..

[84] Tanaka T., Kikuchi Y. (2001). Origin of cloverleaf of transfer RNA—The double-hairpin model: Implication for the role of tRNA intron and the long extra loop. Viva Origino.

[85] Di Giulio M. (2004). The origin of the tRNA molecule: Implications for the origin of protein synthesis. J. Theor. Biol..

[86] Kanai A. (2015). Disrupted tRNA Genes and tRNA Fragments: A Perspective on tRNA Gene Evolution. Life.

[87] Burton Z.F. (2020). The 3-Minihelix tRNA Evolution Theorem. J. Mol. Evol..

[88] Du X., Li Y., Xia Y.L., Ai S.M., Liang J., Sang P., Ji X.L., Liu S.Q. (2016). Insights into Protein-Ligand Interactions: Mechanisms, Models, and Methods. Int. J. Mol. Sci..

[89] Miyazawa S., Jernigan R.L. (1996). Residue-residue potentials with a favorable contact pair term and an unfavorable high packing density term, for simulation and threading. J. Mol. Biol..

[90] Miyazawa S., Jernigan R.L. (1985). Estimation of Effective Interresidue Contact Energies from Protein Crystal-Structures—Quasi-Chemical Approximation. Macromolecules.

